# A Pathogenic Nematode Targets Recognition Proteins to Avoid Insect Defenses

**DOI:** 10.1371/journal.pone.0075691

**Published:** 2013-09-30

**Authors:** Duarte Toubarro, Mónica Martinez Avila, Rafael Montiel, Nelson Simões

**Affiliations:** 1 IBB/CBA and CIRN/Departamento de Biologia, Universidade dos Açores, Ponta Delgada, Portugal; 2 Laboratorio Nacional de Genómica para la Biodiversidad, Centro de Investigación y de Estudios Avanzados del Instituto Politécnico Nacional (CINVESTAV-IPN), Irapuato, Guanajuato, Mexico; German Primate Center, Germany

## Abstract

*Steinernema* *carpocapsae* is a nematode pathogenic in a wide variety of insect species. The great pathogenicity of this nematode has been ascribed to its ability to overcome the host immune response; however, little is known about the mechanisms involved in this process. The analysis of an expressed sequence tags (EST) library in the nematode during the infective phase was performed and a highly abundant contig homologous to serine protease inhibitors was identified. In this work, we show that this contig is part of a 641-bp cDNA that encodes a BPTI-Kunitz family inhibitor (Sc-KU-4), which is up-regulated in the parasite during invasion and installation. Recombinant Sc-KU-4 protein was produced in *Escherichia coli* and shown to inhibit chymotrypsin and elastase activities in a dose-dependent manner by a competitive mechanism with K_i_ values of 1.8 nM and 2.6 nM, respectively. Sc-KU-4 also inhibited trypsin and thrombin activities to a lesser extent. Studies of the mode of action of Sc-KU-4 and its effects on insect defenses suggest that although Sc-KU-4 did not inhibit the activation of hemocytes or the formation of clotting fibers, it did inhibit hemocyte aggregation and the entrapment of foreign particles by fibers. Moreover, Sc-KU-4 avoided encapsulation and the deposition of clotting materials, which usually occurs in response to foreign particles. We show by protein-protein interaction that Sc-KU-4 targets recognition proteins of insect immune system such as masquerade-like and serine protease-like homologs. The interaction of Sc-KU-4 with these proteins explains the ability of the nematode to overcome host reactions and its large pathogenic spectrum, once these immune proteins are well conserved in insects. The discovery of this inhibitor targeting insect recognition proteins opens new avenues for the development of *S* *. carpocapsae* as a biological control agent and provides a new tool to study host-pathogen interactions.

## Introduction

*Steinernema* *carpocapsae* is an entomopathogenic nematode (EPN) that is currently used to control insect pests, owing to its high virulence against a wide variety of insects [1]. The virulence of *S* *. carpocapsae* is mainly attributed to the ability the infective juvenile has to overcome insect defenses and to the symbiotic bacteria it carries into the parasitized insect, which releases toxic factors [2,3].

Insects are equipped with a system of pathogen recognition receptors and effectors that enables them to resist a wide variety of pathogens [4]. Pathogen receptors are found as soluble proteins in body fluids and on the cellular surface, like Toll receptor, and the effectors are composed of cellular and humoral components that cooperate to neutralize invasive organisms [5,6]. A complex reaction of encapsulation takes place when large foreign bodies such as EPNs are encountered [7]. In the encapsulation are participating soluble proteins from the haemocoel, proteins released from activated hemocytes and the hemocytes themselves [4]. This process involves three main events: cell activation, clot formation and activation of phenoloxidase [4,8]. Hemocytes activation is triggered within minutes of pathogen exposure with cells becoming adherent to each other and to the foreign surface [9,10]. The clot formation involves the activation of soluble proteins in the hemocoel, such as transglutaminase, lipophorin, hexamerins, and fondue and proteins derived from hemocytes, for instance hemolectin and tiggrin, that lead to the clotting of hemolymph forming a network of fibers that bind together to isolate the foreign body [11,12]. In the presence of foreign agents a series of proteolytic enzymes are activated leading to the processing of the zymogen prophenoloxidase (PPO) into its active form phenoloxidase (PO). Phenoloxidase produces indole groups, which are polymerized to melanin and subsequently deposited in entrapped foreign body [13]. The three systems work together leading to the formation of hard clots that efficiently protect from invasive pathogens [14].

To escape host defenses EPNs have developed passive and active mechanisms. The passive mechanisms usually mimic the host components to evade detection, whereas in the active process the pathogen actively destroys the host defense effectors [7]. Surface coating proteins that participate in the evasion of the host immune system were identified in *S* *. glaseri* , *S.* feltiae and *H* *. bacteriophora* [15-17] and *S* *. carpocapsae* and *S* *. feltiae* were shown to destroy insect immune effectors namely antibacterial peptides [18,19]. Despite the ability of these nematodes to counteract insect defenses, the pathogenicity of EPNs against a particular insect is usually thought to result from an “arms race” between the EPN and the insect [20,21]. Moreover, there are several reports describing the immune reactions of insects against EPNs [22]. For example, a small part of the *Pseudaletia* *unipuncta* larvae infected with *S* *. carpocapsae* reacts through a cellular encapsulation mechanism [23], the diptera Tipula *oleracea* reacts via a humoral encapsulation mechanism against *S* *. feltiae* [24], and *Drosophila melanogaster* recognizes *H* *. bacteriophora* through transglutaminase, an important component of clot system [25].

The pathogenicity of parasitic nematodes is known to be essentially modulated by the nematode’s secreted and excreted products (ESPs), which are active against effectors of the host’s immune system [26]. Many of these active ESPs are proteases and protease inhibitors. For example, the nematode *Ancylostoma ceylanicum* produces a serine protease inhibitor that prevents the activation of neutrophils [27], the infective stages of *Schistosoma mansoni* and *Brugia malayi* release serpins that protect the parasites during inflammation by inhibiting neutrophil elastases [28,29], and *Trichuris* *suis* releases an inhibitor of trypsin and chymotrypsin that modulates neutrophil proteases [30].

In ESPs of *S* *. carpocapsae* , a few serine proteases were shown to participate in host immune evasion by destroying the insect’s cellular and humoral defenses [31-34]. Furthermore, the analysis of an EST library obtained from *S* *. carpocapsae* during the parasitic phase allowed the detection of a highly abundant contig with homology to the serine protease inhibitors of parasitic nematodes, a few of them shown to participate in host immune modulation [35]. To understand the role of this putative protease inhibitor, we cloned and sequenced the full cDNA and show that it encodes a BPTI-Kunitz family inhibitor. Recombinant protein produced in *E. coli* was characterized, and the effects on insect defenses were determined. Our results suggest that this inhibitor binds to key recognition proteins in the insect, thereby impairing cellular aggregation, modifying clotting fiber arrangements and partially preventing PPO activation, all of them allowing the EPN to avoid encapsulation.

## Materials and Methods

### Nematodes and insects

Infective juveniles of the *S* *. carpocapsae* Breton strain were produced in the larvae of the moth *Galleria* *mellonella* [36], harvested in a White trap [37] and stored in tap water at 10°C for 1–2 months before use.

### Full-length cDNA and genomic DNA

The full-length Sc-KU-4 cDNA was generated using the primers KU-4'Rf (5'-TTTGGTTCCATCTGGGCAAGTGTC-3') and KU-4'Rr (5'-CGCTGTATTGATCGTTTGCTG-3') designed based on an EST expressed in the parasitic stage of *S*. *carpocapsae* (GenBank accession number GR977821). RNA was isolated from nematodes in the parasitic stage as described [35], and the first-strand cDNA was synthesized using a Superscript III reverse transcriptase kit (Invitrogen) and an oligo (dT) primer. The full-length cDNA was produced by rapid amplification of cDNA ends (RACE) using the SMART RACE cDNA Amplification Kit (Clontech-Takara) and the KU-4'Rf and KU-4'Rr primers. The reaction mixture contained 50 ng of cDNA, 2.5 µl of 10x PCR buffer, 10 mM of each dNTP, 0.4 µM of each primer, 1.6 mM MgCl_2_ and 0.2 µl of Taq DNA polymerase in a final reaction volume of 25 µl. The amplification protocol consisted of an initial heating step at 94°C for 3 min, followed by 30 cycles of 94°C for 30 s, 55°C for 30 s and 72°C for 30 s, with a final extension cycle at 72°C for 3 min. Amplified cDNA fragments were TA-cloned into the vector pCR4-TOPO using the TOPO TA Cloning Kit (Invitrogen) and sequenced by Stabvida (Oeiras, Portugal). Full-length cDNA was obtained by aligning the fragments using the Bioedit software package.

Genomic DNA was extracted from nematode parasitic stage and amplified by PCR using flanking primers (Fwd 5'CGCTGTATTGATCGTTTGCTG3' and Rev 5’ CAATCACCTCTGACTTCCAC3’) designed to target the full-length cDNA sequence. The amplification conditions were as follows: 94°C for 3 min, followed by 30 cycles of 94°C for 30 s, 55°C for 30 s and 72°C for 30 s, with a final extension at 72°C for 3 min. The 741-bp PCR product was isolated, cloned into the aforementioned TOPO-TA vector (Promega) and transformed into *E. coli* DH5α cells, and DNA inserts of positive clones were confirmed by sequencing. The exon-intron boundaries were determined by comparing cDNA with gDNA.

### In silico analysis of sc-KU-4

Sc-KU-4 full-length cDNA was used as a BLAST search query (http://ncbi.nlm.nih.gov/blast), and sequence alignments were created with ClustalW (http://www.ebi.ac.uk/clustalw). Protein motifs were predicted using SMART (http://smart.emblheidelberg.de), and the signal peptide was identified by SignalP 3.0 (http://www.cbs.dtu.dk/services/SignalP). For phylogenetic reconstruction, sequences were aligned with Mega5 (http://www.megasoftware.net/mega4/index.html), and the phylogenetic tree was constructed with maximum likelihood using PhyML (www.atgc-montpellier.fr/phyml/). Robustness was assessed by the bootstrap method (100 pseudoreplicates). The 3D structure prediction was obtained using the on-line platform I-TASSER (http://zhanglab.ccmb.med.umich.edu/I-TASSER).

### Analysis of gene expression

Sc-KU-4 mRNA levels were determined by quantitative RT-PCR during different stages of nematode development in a parasitized insect. For this purpose, parasitism was generated by exposing *G* *. mellonella* larvae to nematode infective juveniles (IJs) in Petri dishes padded with wet filter paper. The different nematode stages were collected from parasitized insects after inspection for the desired stages. L3 stage nematodes were isolated from the gut and the hemocoel, L4 stage nematodes were isolated from the hemocoel, and adults and L1/L2 stage nematodes were isolated from the body tissues. Approximately 20 specimens were collected from each stage in three separate exposures and homogenized in liquid nitrogen; total RNA was extracted using the RNeasy Micro Kit (Qiagen). Reverse transcription was performed using the Superscript III reverse transcriptase kit (Invitrogen) and an oligo (dT) primer. Data were normalized to 18S rRNA. Sc-KU-4 was amplified with the primers Ku-4Fg (5'- TAC AAC GGG ACT GGA GGC AA -3') and KU-4Rg (5'- TGG TTC CAT CTG GGC AAG TG -3'), and the 18S rRNA with primers 18SF (5'-GCTAATCGGAAACGAAAGTC-3') and 18SR (5'-CATCCACCGAATCAAGAAAG-3') using an ABI 7900 HT Real Time PCR thermocycler (Applied Biosystems). Each reaction consisted of an initial heating step at 95°C for 10 min, followed by 60 cycles of 95°C for 15 s and 60°C for 60 s. Amplifications were performed in triplicate. RT-PCR data were analyzed using the comparative C_T_ method according to the manufacturer’s recommendations. Data were expressed as means ± standard errors. Statistical analysis was carried out by one-way ANOVA followed by Bonferroni multiple comparison tests (SPSS software, version 13.0). Differences were considered significant at P values < 0.05.

### Construction of the expression vector

The full-length *sc-ku-4* coding sequence was amplified using the forward primer containing an NdeI restriction site (5'- CATATGATGTTTCCACTTTTCGAGTTTAAC -3'

) and the reverse primer containing an XhoI site (5'- CTCGAGTTCCTTCGAGCAGCATTTAGC -3'). The 0.8 kb product was purified from a 1.5% agarose gel using the QIAEX II Gel Extraction Kit (Qiagen) and digested with NdeI and XhoI. The fragment was then inserted into the expression vector pET23a (+) to produce the recombinant vector pET23a (+)-sc*-ku-4* in frame with a C-terminal His6X-tag. The vector was introduced into *E*. *coli* C41 (DE3) and Rosetta™ II (DE3) strains. Identity and integrity of the inserts were confirmed by sequencing.

### Expression of recombinant Sc-KU-4

A single colony of each transformed *E. coli* strain was incubated in 5 ml Luria broth (LB) containing 100 µg/ml ampicillin and 34 µg/ml chloramphenicol at 37°C and 250 rpm overnight. These cultures were used to inoculate 20 ml LB with antibiotics as above and were incubated at 37°C until the OD_600_ reached 0.6. Sc-KU-4 expression in both strains was induced under the following conditions: 0.2 mM isopropyl β-D-1-thiogalactopyranoside (IPTG) at 37°C for 3 h, 1 mM IPTG at 37°C for 3 h, 0.2 mM IPTG at 20°C overnight and 1 mM IPTG at 20°C overnight. Cells from the different induction conditions were harvested by centrifugation at 6,500 x g for 15 min, and the pellet was resuspended in 2 ml 0.05 M Tris-HCl (pH 7.4) containing 0.5 M NaCl (TN) and 1 mg/ml lysozyme and frozen overnight at −20°C. After thawing, 1 ml 1 M MgCl_2_ and 600 µl 1 mg/ml DNAse were added and the solution was incubated for 30 min at RT under orbital agitation. Cellular debris was removed by centrifugation at 12,000 x g for 20 min, and the supernatant was collected and analyzed for recombinant protein expression.

### SDS-PAGE and Western blot

Suspensions with recombinant Sc-KU-4 were run on a 10% SDS-PAGE in a Mini-PROTEAN II gel system (Bio-Rad), and proteins were stained with colloidal Coomassie. For Western blot analysis, proteins in the gel were electro-blotted onto a PVDF membrane using a mini Trans-Blot Cell (Bio-Rad). The membrane was blocked with 0.01 M Tris-HCl (pH 7.5) containing 0.1 M NaCl, 5% (w/v) BSA and 0.05% (v/v) Tween-20 for 30 min at RT, incubated with anti-His6X-tag peroxidase-conjugated mouse antibody diluted in blocking solution (1:5,000) for 2 h at RT and then washed 3 times for 10 min with 0.01 M Tris-HCl (pH 7.5), 0.1 M NaCl. Antibodies were detected by incubation with the tetramethylbenzidine peroxidase substrate (Sigma-Aldrich) according to the manufacturer’s instructions.

### Production, refolding and purification of Sc-KU-4

Recombinant Sc-KU-4 was produced in transformed *E. coli* Rosetta (DE3) cells in 2 L of LB medium, and expression was induced with 0.2 mM IPTG followed by a 3 h incubation at 37°C. Cells were recovered after centrifugation at 6,500 x g for 15 min in 100 ml of TN and 1 mg/ml lysozyme, incubated for 30 min at RT and then frozen overnight. After thawing, the solution was adjusted to 500 ml with TN and centrifuged at 10,000 x g for 15 min. The pellet was resuspended in 1 L of TN plus 1% Triton X-100 and incubated under agitation for at least 4 h at 4°C.

Inclusion bodies were retrieved by centrifugation at 10,000 x g for 20 min at 4°C, followed by solubilization in 6 M urea containing 0.02% β-mercaptoethanol overnight with orbital agitation. Solubilized protein was dialyzed in a cellulose membrane tube with 20 mM Tris-HCl (pH 8) for 48 h at 4°C, followed by two changes in the same buffer. Recombinant protein was captured by nickel chelate affinity chromatography (His-Tag, GE) under the recommended conditions in an AKTA FPLC system (GE Healthcare). Purified recombinant proteins were refolded for 3 h in 50 mM Tris (pH 8) with 300 mM L-arginine, 50 mM NaCl and 5 mM DTT. Folded Sc-KU-4 was applied to a MonoQ column (GE) equilibrated with 20 mM Tris-HCl (pH 8) and eluted on a linear gradient of 1 M NaCl to eliminate minor contaminants. Purity was assessed by 12% SDS-PAGE. The protein concentration was determined using the BCA Kit (Thermo Scientific).

### Sc-KU-4 specificity

The inhibitory activity of Sc-KU-4 was tested against trypsin, α-chymotrypsin and elastase from bovine pancreas and thrombin from bovine plasma (Sigma).

Ten micrograms of purified Sc-KU-4 was added to 2 µg of each enzyme, and the final volume was adjusted to 50 µl with 0.1 M Tris-HCl (pH 7.5) containing 0.1 M NaCl and 1 mM CaCl_2_. After incubation for 15 min at 37°C, the remaining hydrolytic activity was quantified using 1 mM of each specific chromogenic substrate: BApNA, Suc-AAPFpNA, Suc-AAPLpNA and BPVA-pNA (Sigma) for trypsin, α chymotrypsin, elastase and thrombin, respectively. Sc-KU-4 was replaced with buffer in control reactions. The formation of p-nitroaniline was monitored at 405 nm in an ELISA microplate reader (Bio-Rad). The percentage of enzyme inhibition was calculated as follows: % inhibition = [

(total enzyme activity units - residual enzyme activity units)/total enzyme activity units] X 100. IC_50_ values were calculated using Probit (v1.4) based on the percentage of inhibition of enzymatic activity after incubation with increasing concentrations of recombinant Sc-KU-4 (0, 5, 11 and 16 µM) under the conditions described above.

### Kinetic analysis

The fractional activity (velocity of enzyme with Sc-KU-4 / velocity of enzyme without Sc-KU-4) was plotted against the ratio of inhibitor / enzyme concentrations, and the *x*-axis intercept as a value for the stoichiometry of inhibition (SI) was determined by linear regression analysis. The inhibition constant (K_i_) of Sc-KU-4 for α-chymotrypsin and elastase was estimated using a Dixon plot. Recombinant Sc-KU-4 (0, 5, 11 and 16 µM) was incubated with 5 µM of each enzyme for 10 min at 37°C; then, 0.05, 1 and 2 mM of Suc-AAPFpNA or Suc-AAPLpNA was added to the α-chymotrypsin and elastase reactions, respectively. The remaining hydrolytic activity was monitored as described above. A single regression line for each concentration of substrate was obtained, and the K_i_ was calculated from the intersection of the three lines. The inhibitory mechanism was determined using Lineweaver-Burk plots. Regression lines for α-chymotrypsin and elastase were produced using the inverse of the initial rate for each Sc-KU-4 concentration plotted against the inverse of the concentration of the substrate.

### Isolation of plasma proteins interacting with Sc-KU-4

*G* *. mellonella* hemolymph was collected by a ventral pro-leg puncture of the 4th instar larvae and was drained into an anticoagulant solution (Ringer without CaCl_2_ and with added 20 mM EDTA). Hemocytes were removed by centrifugation at 1,500 x g for 3 min at 4°C, and plasma was recovered and diluted (5:1) in the anticoagulant solution plus 0.1% polyvinylpyrrolidone.

Sc-KU-4 was immobilized on CNBr-activated Sepharose beads 4B (Sigma) according to the manufacturer’s instructions and then incubated with 50 mg of plasma proteins in 2 ml of 20 mM Tris-HCl (pH 8) for 3 h at 4°C with orbital agitation. BSA-coated beads were used as controls. After the reaction, beads were packed onto a 0.7 × 0.5 cm column coupled to an AKTA FPLC system (GE Healthcare). Unbound material was washed through the column with binding buffer, followed by 5 column volumes of 20 mM Tris-HCl(pH 8), 0.5 M NaCl. The remaining bound proteins were eluted with 50 mM glycine (pH 3). Eluted proteins were dialyzed with a centricon (MWCO 10 kDa, Millipore), suspended in rehydration buffer (9.8 M Urea, 4% (w/v) CHAPS, 2 mM TBP, 1% (v/v) ampholytes pH 3-10) and applied to a 7 cm IPG strip (Bio-Rad) for 2DE. After active rehydration for 12 h at 20°C, proteins were focused in a Protein IEF Cell (Bio-Rad). Prior to the second dimension, the IPG strip was equilibrated with 130 mM DTT and 135 mM iodoacetamide. SDS-PAGE and protein staining were performed as above.

### MALDI-MS/MS

For MALDI-MS/MS analysis, proteins were digested in-gel with trypsin, purified and concentrated as previously described [33]. The m/z spectra were acquired in a 4700 Proteomics Analyzer MALDI-TOF/TOF (Applied Biosystems) in both MS and MS/MS mode. Protein identification was achieved using MASCOT (www.matrixscience.com) to search the UniProtKB database (downloaded on 10/06/2012).

### Analysis of phenoloxidase activity

Plasma was prepared as described above and PO activity quantified as previously described [38] with minor modifications. Forty microliters of plasma solution was added to 10 µl of Sc-KU-4 (3 µM/µl) and incubated for 5 min on ice. In controls, Sc-KU-4 was replaced with anticoagulant solution. The samples were then activated with 5.5 µl (10 µg/ml) of lipopolysaccharide (LPS *E. coli* O55:B5, Sigma - Aldrich) dissolved in 50 mM Tris-HCl (pH 7.5), 0.1 M NaCl and incubated 15 min at 25°C. Activated plasma was centrifuged at 5,000 x g for 5 min at RT, the supernatant was recovered and supplemented with 20 µl 15 mM L-DOPA (Sigma) dissolved in 20 mM Tris-HCl (pH 7.5), and the reaction was incubated for 15 min at 37°C. The reaction was monitored at 490 nm using an ELISA microplate reader (Bio-Rad). PO activity was measured as the relative change in OD at reaction time points of 0 and 15 min. Experiments were carried out in triplicate.

### Analysis of hemolymph coagulation and encapsulation assay

The analysis of clotting fibers was carried out on a slide by adding Sc-KU-4 (30 µM) to 200 µl of plasma, incubated for 30 min in a humidified chamber at RT and then activated with LPS (10 µg/ml). In the controls, Sc-KU-4 was replaced with the anticoagulant solution. The incorporation of melanin into clots was tested by carefully aspirating the supernatant with a fine tip. Adherent materials were then visualized and photographed by light microscopy. To check for the formation of clotting fibers, plasma drops were prepared and incubated as described and were fixed with 2% glutaraldehyde in 10 mM phosphate buffer (PB) (pH 7) at 4°C for 15 min. The fixative was removed gently by washing with 10 mM PB and replaced with 70% ethanol. Samples of the floating clot strands were carefully transferred to two new slides, one for phase contrast observation and the other for peanut agglutinin-FITC conjugated (PNA-FITC) staining (Sigma) (10 mg/ml) in PB and observed at 480 nm by a fluorescence microscope (Zeiss). Hemocyte aggregation and activation were produced in elicited hemolymph according to a previously described method [10]. Hemocyte aggregates were inspected using an optical microscope at 50X magnification and data was obtained from five optical fields of three replicates. Statistical analyzed was performed using SPSS software, version 13.0. In the encapsulation assay, beads of Sephadex G200 were soaked with Sc-KU-4 for 10 min, added to plasma activated with LPS, incubated for 30 min in a humidified chamber at RT and then fixed with 2% glutaraldehyde. In a second assay of encapsulation, the plasma was first incubated with Sc-KU-4 for 30 min at RT, then mixed with Sephadex G200 beads, incubated for 30 min and fixed with 2% glutaraldehyde as described above. Fixed materials were processed for SEM as described elsewhere [34]. Experiments were carried out in triplicate.

## Results

### Molecular Characterization of Sc-KU-4

A full-length 641 bp cDNA was produced using primers designed based in the EST sequence predicted for a serine protease inhibitor of *S* *. carpocapsae* . After cloning and sequencing, a single open reading frame of 555 bp was identified and submitted to NCBI with the accession number HM586101. This ORF was denoted Sc-KU-4, and has a 5´ UTR with an “*ATG*” start codon and an 86-bp 3´ UTR in which a stop codon is followed by a non-canonical polyadenylation signal “*CATAAA*” and a poly (A) tail. This cDNA codes for a protein with 184 amino acids, including a signal peptide of 19 residues and a single Kunitz domain beginning at aa 27 and ending at aa 77 (NCBI accession ADQ44000). The putative mature protein had a MM of 20,418.16 Da and a pI of 5.07.

### Genomic organization

A single 741 bp fragment corresponding to the genomic DNA of Sc-KU-4 was amplified using primers flanking the 5’ and 3’ regions of the ORF and was sequenced and submitted to NCBI (Accession number - HQ328060). The alignment of gDNA and cDNA suggests that the *sc-ku-4* gene has five exons containing 43, 72, 123, 140, and 175 bp, which are coded for by residues 1-43, 92-163, 211-333, 380-520 and 566-741, respectively, all flanked by the typical splice signals AG/GT (see hyperlink http://www.ncbi.nlm.nih.gov/nuccore/312192649?report=graph). The first exon encodes the signal peptide and exons II and III encode the Kunitz domain.

### Sequence homology, phylogeny and predicted structure

**Figure 1:**
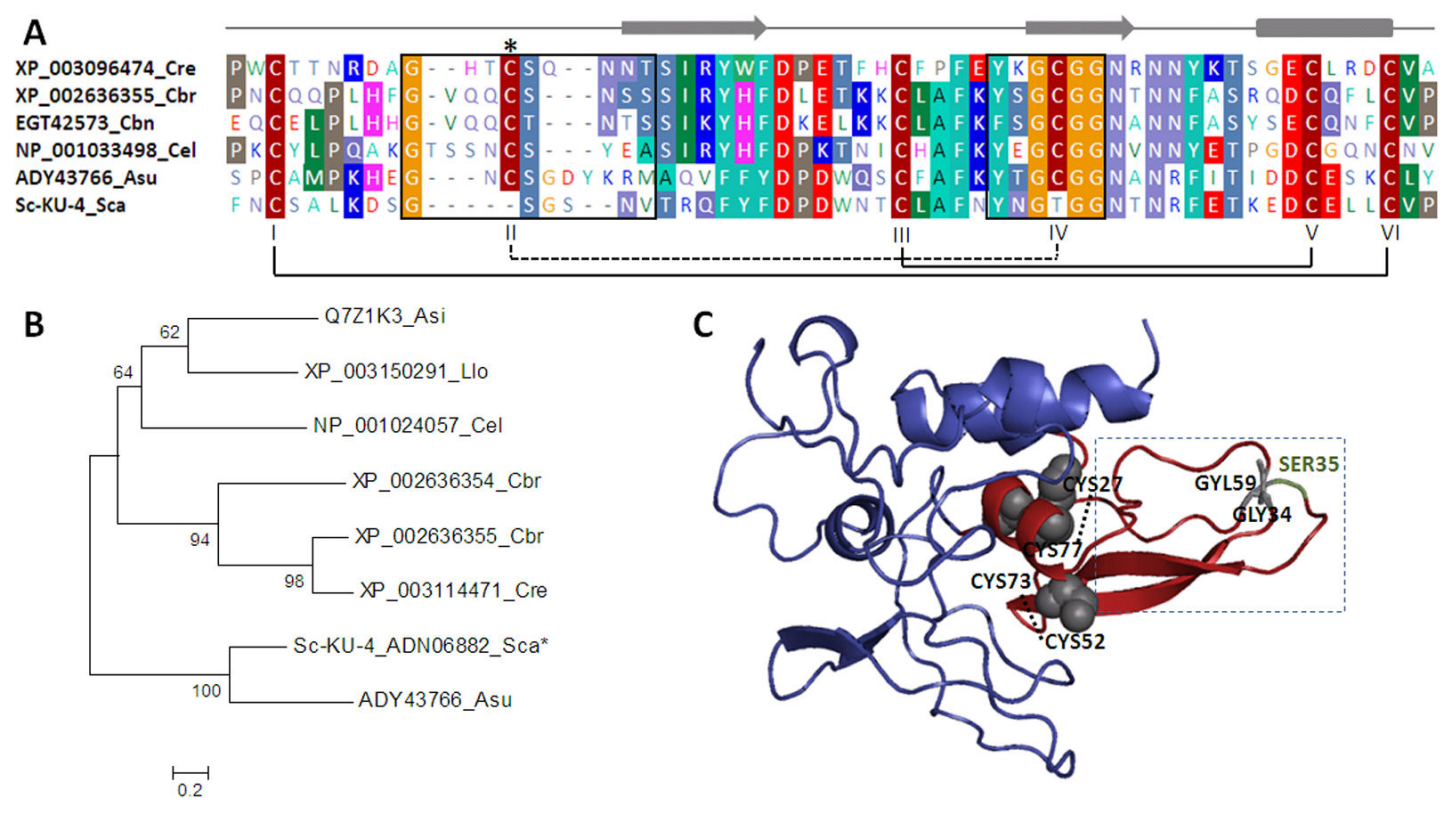
Sequence, phylogeny and structural analysis of Sc-KU-4. **A**: Amino acid sequence alignment of the Kunitz domain in Sc-KU-4 with the homologous domains of other nematode Kunitz-like inhibitors. Conserved positions are shaded; the solid lines indicate the conserved disulfide bridges, and the discontinuous line indicates the missing CysII–CysIV bridge in Sc-KU-4. **B**: Phylogenetic analysis showing Sc-KU-4 closes to other nematode Kunitz-like inhibitors forming a separate cluster with *A*. *suum*. **C**: Sc-KU-4 structure prediction based on multiple-threading alignments showing the Kunitz domain exposed on the molecular surface in a characteristic tertiary fold of three antiparallel β-strands followed by one α-helix. Cysteine residues responsible for reactive loop stabilization and glycine residues that replace CysII–CysIV are shown in gray. The discontinuous line box highlights the amino acids present in reactive loops. The P1 position is shown in green. Asi: *Anisakis* *simplex*; Asu: *Ascaris* *suum*; Cbn: *Caenorhabditis* *brenneri*; Cbr: *C*. *briggsae*; Cel: *C*. *elegans*; Cre: *C*. *remanei*; Llo: *Loa* *loa*; Sca: *S*. *carpocapsae*.

The Sc-KU-4 sequence has identity with secreted Kunitz-like serine protease inhibitors of the parasitic nematodes *Ascaris* *suum* (45%), *Loa* *loa* (35%) and *Anisakis* *simplex* (30%) and also has homology with Kunitz domains from the free-living nematodes *Caenorhabditis* *remanei* (32%), *C* *. briggsae* (30%) and *C. elegans* (30%). All of these nematode Kunitz inhibitors shared a similar molecular architecture with an N-terminal signal peptide followed by a single Kunitz domain, except for the inhibitor from *A* *. suum* , which presented two Kunitz domains in tandem. Multiple sequence alignment showed that Sc-KU-4 has a BPTI-Kunitz family domain with 60 amino-acid residues located at the NH terminus (Figure 1A). The Sc-KU-4 Kunitz domain contained the conserved residues Gly34, Tyr57, Phe55 and Gly59 and four of the six well-conserved cysteine residues. Therefore, it was possible that the CysI - CysVI and CysIII – CysV disulfide bridges were formed (Cys27-Cys77 and Cys52-Cys73) but that the CysII – CysIV bridge was missing. The positions of CysII and CysIV in the canonical Kunitz domain were occupied by Gly36 and Gly61 in Sc-KU-4. The putative primary binding site (P1 residue) in Sc-KU-4 was predicted to be occupied by a serine (Ser37), as in the *A* *. suum* *,* *C. remanei* *, C. elegans and* *C* *. briggsae* homologs.

The phylogenetic analysis revealed that Sc-KU-4 clustered with other nematode Kunitz-like inhibitors, which diverged early from the canonical bovine BPTI-Kunitz inhibitor. In the nematode cluster, Sc-KU-4 formed a group with the parasitic nematode *A* *. suum* , which was separated from the group formed by the *Caenorhabditis* free-living species and the parasites *A* *. simplex* and *L. loa*. These conclusions are supported by high bootstrap values (100%) in a Nearest-Neighbor-Interchange tree (Figure 1B).

A predicted 3D model for Sc-KU-4 was generated using the I-TASSER server, with an estimated model accuracy of 0.72 ± 0.10 (TM-score). Under this model, the active domain of Sc-KU-4 was predicted to preserve the three antiparallel β-strands followed by one α-helix, the characteristic tertiary fold of the Kunitz domain. The preservation of such structure is probably due to the Van Der Waals interactions established among the glycine residues that replaced CysII and CysIV, thus allowing for the stabilization of the recognition loops (Figure 1C). The two loops originated by β-strand twist are particularly important for inhibitor recognition. The first recognition loop contained residues 32-GSGSNVTV-38 (P4 - P4´), and the second contained residues 57-YNGTG-61. In this model, the scissile bond site was predicted to be exposed on the protein surface.

### Bacterial transformation and production of recombinant Sc-KU-4

The *Sc-ku-4* coding sequence was inserted into the expression vector pET23a in frame with a C-terminal His6X-tag. Recombinant Sc-KU-4 was produced only in inclusion bodies, and the highest recombinant concentration (74 mg/L) was obtained in the *E. coli* host strain Rosetta (DE3) induced with 0.5 and 1 mM of IPTG for 3 h at 37°C (Figure S1). Sc-KU-4 was isolated in a single step by Ni^2+^ affinity chromatography, folded and purified using Mono Q chromatography. After purification, one band with the expected mass of approximately 20 kDa appeared on an SDS-PAGE gel and was detected with an anti-His(C-term)-HRP antibody. MS/MS spectrometry confirmed that this protein was Sc-KU-4 with a significant score of 306 (protein score probability limit with P < 0.05 is 85) and 34% coverage.

### Sc-KU-4 exhibits a competitive inhibitory mechanism

**Figure 2:**
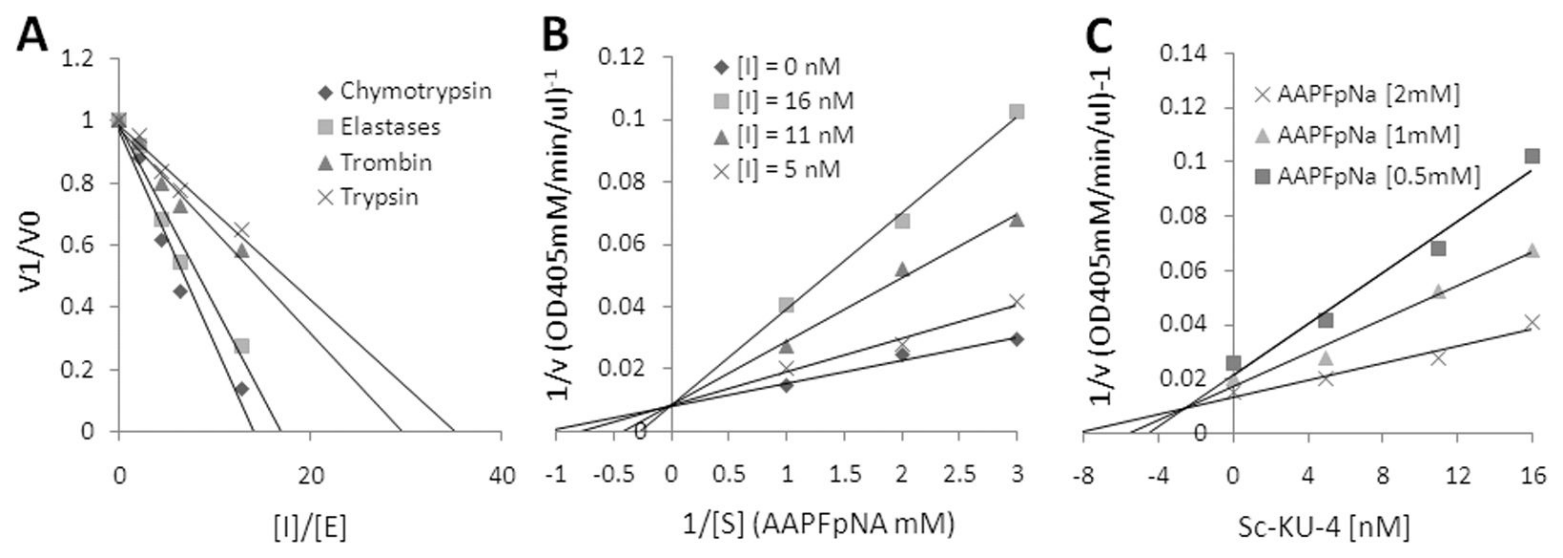
Recombinant Sc-KU-4 inhibits a broad range of trypsin-like enzymes. **A**: Stoichiometry of inhibition of Sc-KU-4 determined against α-chymotrypsin, elastase, thrombin and trypsin. **B**: The competitive inhibitory mechanism of Sc-KU-4 revealed by Lineweaver-Burk reciprocal plots. **C**: The highest Sc-KU-4 K_i_ of 2.6 nM was obtained for α-chymotrypsin.

Recombinant Sc-KU-4 strongly inhibited the hydrolytic activity of α-chymotrypsin and elastase with IC_50_ of 6.3 nM and 8.6 nM. A weak inhibitory effect on the activity of thrombin and trypsin was also detected. The stoichiometry of inhibition (SI) for Sc-KU-4 was 14.26, 16.94, 29.57 and 35.25 for α-chymotrypsin elastase, thrombin and trypsin, respectively (Figure 2A). Sc-KU-4 displayed a competitive inhibitory mechanism, as shown by Lineweaver-Burk reciprocal plots (Figure 2B). K_i_ values of 1.8 nM and 2.6 nM were determined against α-chymotrypsin and elastase, respectively (Figure 2C).

### Invasive nematodes express the highest levels of sc-*ku-4*

**Figure 3:**
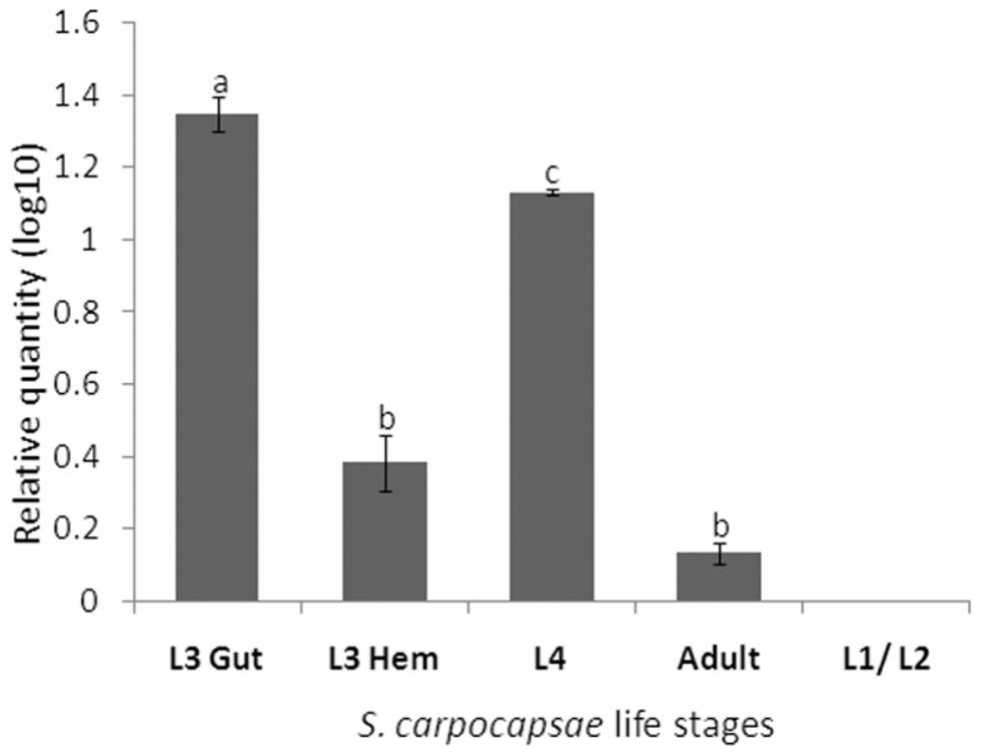
Transcript quantification of Sc-KU-4 during successive stages of the *S. carpocapsae* life cycle reveals an up-regulation during the invasive phases. Sc-KU-4 relative expression was determined by quantitative RT-PCR using 18S as an endogenous control. Nematodes in each phase were collected from parasitized *G*. *mellonella* larvae: L3-gut – third stage juveniles collected inside the gut lumen at 6 h post-exposition (HPE); L3-hem – third stage juveniles collected in the hemocoel at 12 HPE; L4 – fourth stage juveniles collected in the hemocoel 36 HPE; Adults – males and females collected in the hemocoel at 56 HPE; L1/L2 – a pool of first and second stage juveniles collected in the hemocoel at 72 HPE. Bars represent standard deviations from three independent replicates. Different letters indicate significant differences (p < 0.05).

Transcript levels of *sc-ku-4* were measured in all of the nematode stages of development in parasitized *G* *. mellonella* larvae, and in the L3 at two time points. L3 nematodes collected in the digestive tract of parasitized insects and L4 nematodes already in the hemocoel of living insects expressed the highest levels of *sc-ku-4* mRNA. Remarkably, significantly less (P < 0.05) *sc-ku-4* mRNA was expressed by L3 nematodes just after hemocoel invasion. Adult nematodes collected from insect cadavers (approximately 56 HPE) expressed significantly lower levels of *sc-ku-4* than the larval stages. L1/L2 stage nematodes collected from cadavers (approximately 72 HPE) did not express *sc-ku-4* (Figure 3). These data indicate that nematodes (L3 and L4) present in live insects expressed the highest levels of *sc-ku-4*.

### Sc-KU-4 does not impair hemocyte activation but impairs hemocyte aggregation

**Figure 4:**
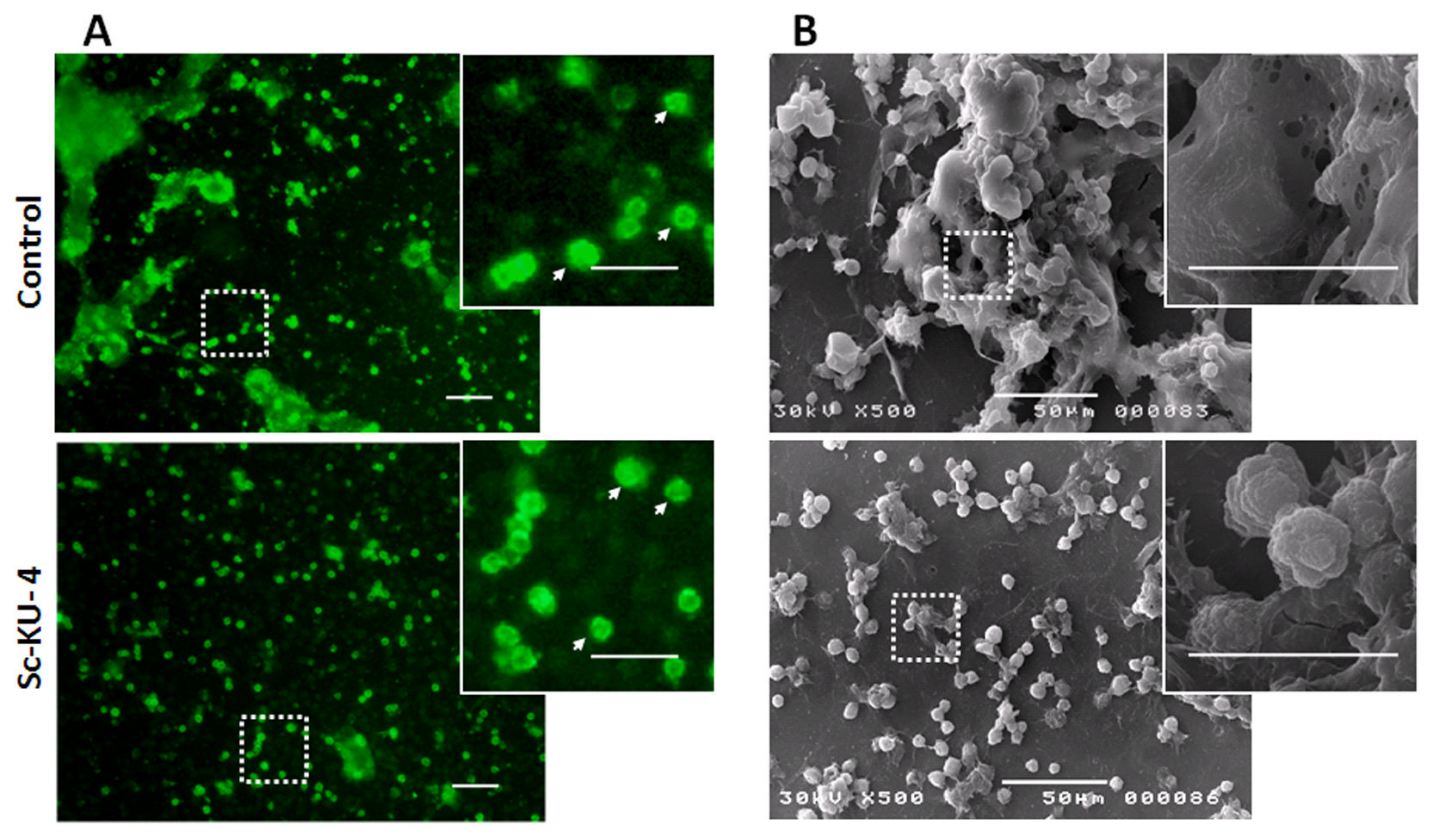
Sc-KU-4 impairs hemocyte aggregation. **A**: Hemolymph stained with peanut agglutinin-FITC conjugated (PNA-FITC) reveal a significant (p < 0.05) less number of aggregates on samples treated with Sc-KU-4 that in control. Also the size of aggregates was significantly (p < 0.05) smaller in treated samples. Magnified boxes highlight activated hemocytes (arrows). **B**: SEM showing hemocytes aggregated in hemolymph activated with LPS whereas in hemolymph treated with Sc-KU-4, the huge majority of hemocytes remained dispersed. Magnified boxes highlight that hemocytes aggregates formed in the control were trapped in a newly formed matrix but in treated samples were not (arrows). Three independent replicates were done.

Sequence homologies and expression profiles of Sc-KU-4 suggest that it should play a role in modulating the host defenses. Therefore, we analyzed the effects of Sc-KU-4 in hemocyte aggregation using the hemolymph of *G* *. mellonella* elicited with LPS. Significant differences (p < 0.05) were observed between the number of hemocyte aggregates formed in the hemolymph treated with Sc-KU-4(5.3 ± 1.5) and in the control (14.5 + 4.4) (Figure 4A). The size of aggregates (measured in the larger diameter) was also significantly different (p < 0.05) in samples treated with Sc-KU-4(66 µm ± 11) and in controls (280 µm ± 27). Because the aggregation of hemocytes requires its activation, we used a previously described method [10] to investigate whether Sc—KU-4 interferes with the activation. In this experiment activated hemocytes were observed in hemolymph treated with Sc-KU-4, similarly to the control, thus indicating that Sc-KU-4 impaired the aggregation of hemocytes but did not impair its activation.

Detailed inspections by SEM consistently showed that the large aggregates of hemocytes formed in the control were trapped in a newly formed matrix whereas they were not in hemolymph treated with Sc-KU-4 (Figure 4B).

### Sc-KU-4 does not impair clot activation but modifies clotting fibers

**Figure 5:**
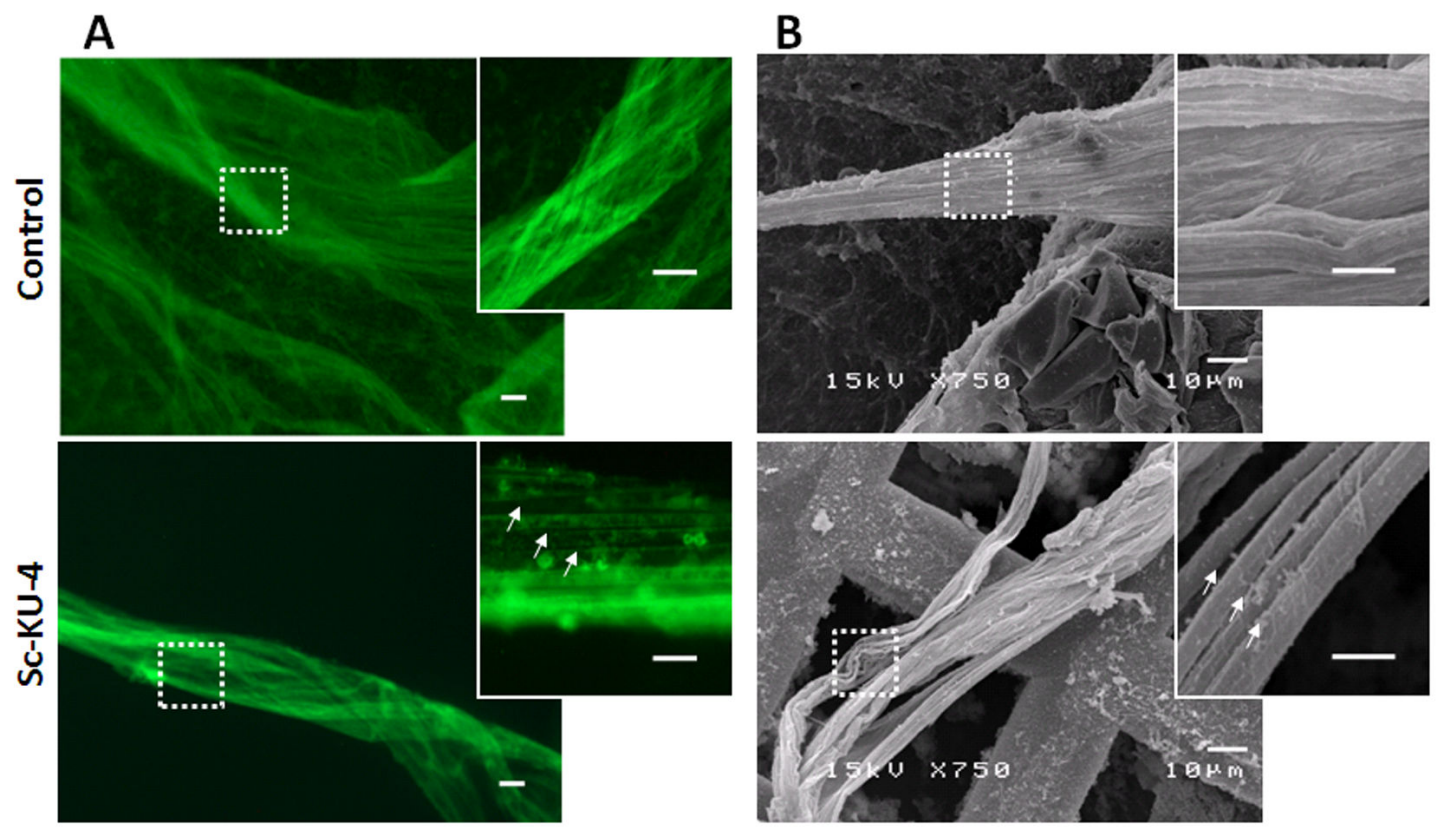
Sc-KU-4 prevents the clotting fibers from forming a stretched mesh. **A**: Clotting fibers observed in activated plasma, after stained with PNA-FITC. In the control a large mesh of fibers was formed whereas in plasma treated with Sc-KU-4, the mesh was less expanded. **B**: Fibers observed by SEM appeared compacted in the control, whereas in treatments with Sc-KU-4 they were individualized. Magnified boxes highlight the individualized fibers (arrows) present in samples treated with Sc-KU-4, in SEM and PNA-FITC.

Clotting fibers were visualized in plasma treated with Sc-KU-4 just as they were in the plasma control after activation with LPS. However, the clotting fibers formed in plasma treated with Sc-KU-4 were less expanded than those formed in the control (Figure 5). A more detailed inspection by SEM showed that clotting fibers in plasma treated with Sc-KU-4 appeared more striated, exhibiting fibbers more individualized than those observed in non-treated plasma.

### Sc-KU-4 does not inhibit PPO activation but slightly decreases PO activity

**Figure 6:**
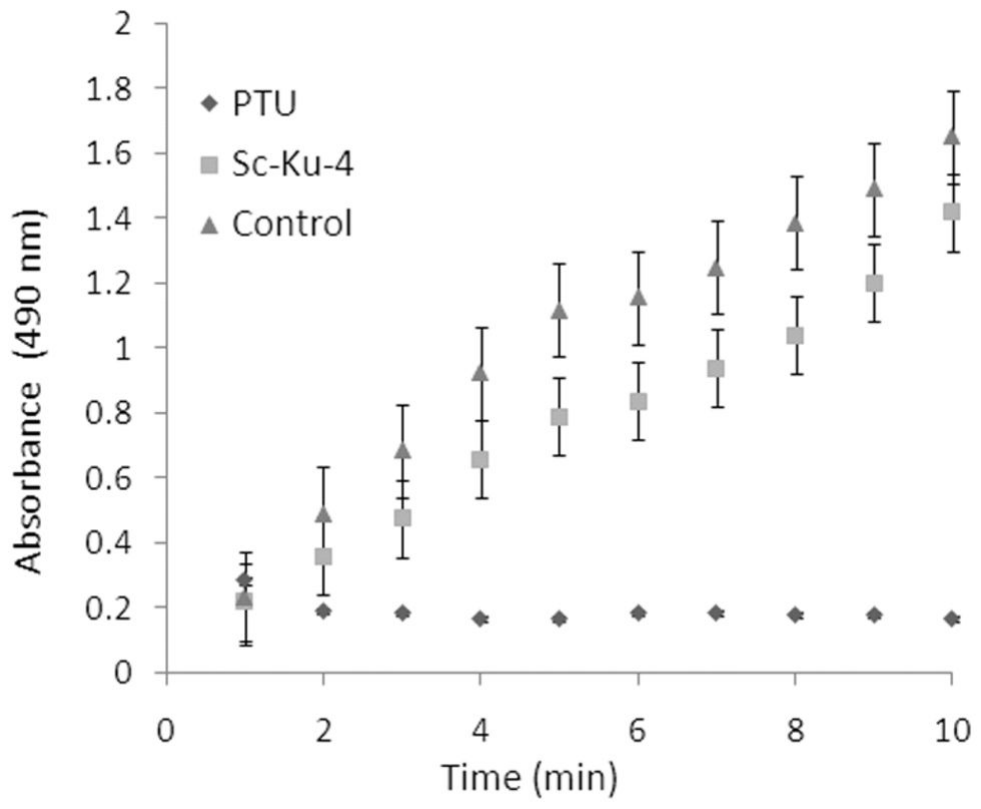
Sc-KU-4 does not inhibit PPO activation, despite the slightly lowered Vmax of PO activity. Vmax of PO in Sc-KU-4-treated plasma was lower than in non-treated plasma, but the final product formed did not differ significantly (P > 0.05) from the control. Each point was plotted from a mean of three replicates. Error bars are shown indicating standard errors.

Our assays showed that Sc-KU-4 did not inhibit PPO activation; however, the kinetics of PO activity suggested that the Vmax was slightly lower in plasma treated with Sc-KU-4 than in non-treated plasma. Furthermore, the final product of the reaction did not differ significantly (P > 0.05) in both assays (Figure 6).

### Sc-KU-4 impairs the aggregation of foreign bodies and the deposition of clotting materials

**Figure 7:**
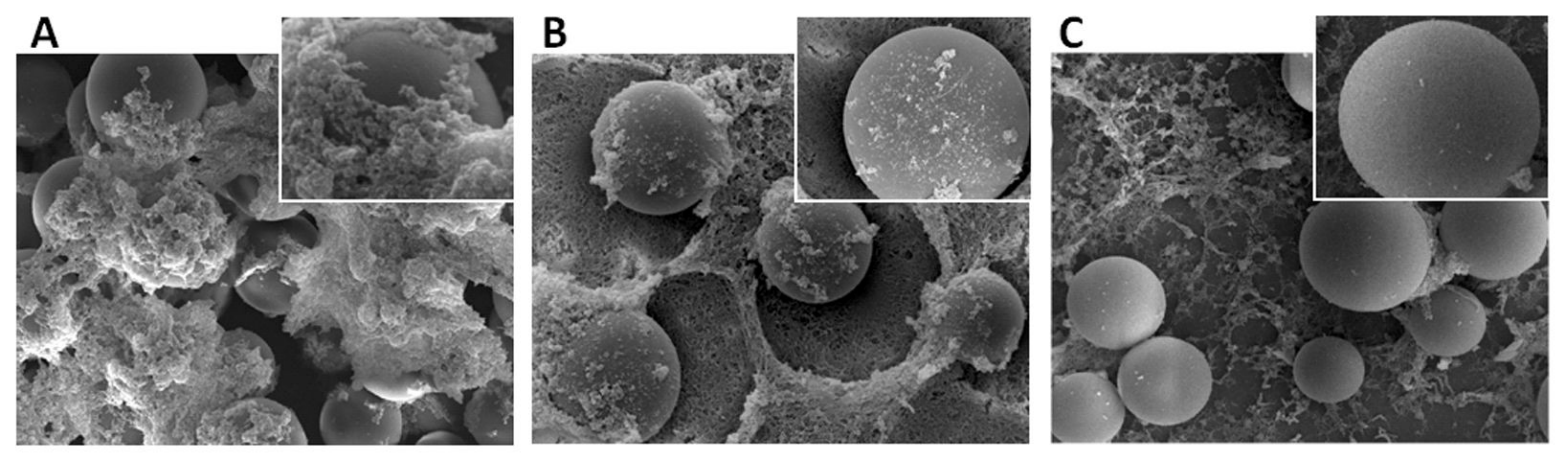
Sc-KU-4 treatment prevents encapsulation of foreign particles. **A**: Non treated Sephadex beads were attached and entrapped by clotting fibers forming aggregates, as observed in the scanning electron micrographs. **B**: Sephadex beads soaked with Sc-KU-4 remained individualized in the plasma and were not fully encapsulated, forming halos (arrows). **C**: A general inhibition of clotting reaction was observed in plasma treated with Sc-KU-4 with a reduction in the number of fibers formed (head arrows) and beads not displaying adhesion of clotting material but instead staying with a slick surface.

To determine if Sc-KU-4 might protect a foreign body from the host response, we added beads soaked in Sc-KU-4 to *G* *. mellonella* plasma activated with LPS. Non-treated beads aggregated as expected but beads treated with Sc-KU-4 did not aggregate and remained individualized. Moreover, treated beads were surrounded by a halo free of clotting mesh, whereas non-treated beads were entrapped by clotting materials. The relevance of Sc-KU-4 in avoiding the recognition of foreign bodies was evidenced also by the distribution of the halos around the beads. The halo was localized in the side of the bead the liquid runoff, whereas in the opposite the bead was covered by clotting material (Figure 7B).

In another assay, plasma was incubated with Sc-KU-4 for a few minutes and beads were subsequently added. Despite the formation of a light mesh of fibers in this assay, the beads remained individualized with a completely polished surface. These results contrasted with those observed for the controls in which beads were aggregated and enclosed in newly formed clotting materials (Figure 7C).

### Sc-KU-4 targets serine protease homologs of *G* *. mellonella* plasma

**Figure 8:**
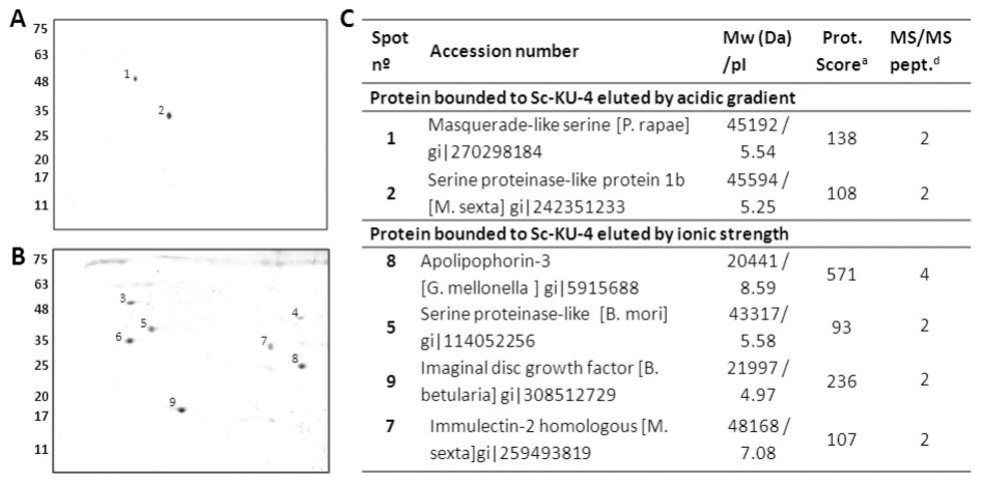
Sc-KU-4 targeted host pattern recognition proteins. Insect plasma proteins were captured by recombinant Sc-KU-4 after performing a pull-down protein, eluted and analyzed by 2DE. **A**: Two proteins were detected in the eluate obtained from the Sc-KU-4-coupled beads with an acidic gradient. **B**: Seven proteins were detected in the eluate obtained with a salt gradient. Isofocalization were performed on 3-10 NL IPG strips, and the second dimension was run on a 12% SDS-PAGE gel. Proteins were stained with colloidal Coomassie blue. MM is shown in kDa. **C**: Sc-KU-4 targeted proteins identified by MS/MS. a: Protein score probability limit (P < 0.05) of 85; b: Only peptides with confidence intervals above 95% were considered; c: Sequence coverage of identified peptides; d: Number of peptides identified by MS/MS.

To determine which proteins in insect plasma were targeted by Sc-KU-4, we conducted a pull-down protein/protein assay using recombinant Sc-KU-4 as bait. A differential elution was performed to recover proteins according to binding forces. Two proteins strongly bounded to Sc-KU-4-coupled beads were eluted using a low pH (pH 3) buffer, detected by 2DE and identified by MS/MS with significant scores in Lepidoptera (Figure 8 and Table S1). One was identified as a homolog of the masquerade-like serine protease (gi:270298184) of *Pieris* *rapae* and another as the serine protease-like 1b (gi:242351233) of *Manduca sexta*, both of which belong to the family of serine protease homologs (SPHs). Among the proteins weakly bounded with Sc-KU-4-coupled beads, eluted by ionic strength, four displayed significant scores by MS/MS: a homolog of a serine proteinase-like protein (gi:114052256) of *Bombyx mori*; a homolog of apolipophorin-3 (gi:5915688) of *G* *. mellonella* ; a homolog of immulectin-2 (gi:259493819) of *M. sexta*; and a homolog of imaginal disc growth factor (gi:308512729) of *Biston* *betularia* . Three other proteins eluted by ionic strength had no homology to any known proteins in NCBI.

## Discussion

*S* *. carpocapsae* is an entomopathogenic nematode that can overcome host defenses by mechanisms that are not well understood [19]. In this work, we show that the nematode expresses a serine protease inhibitor during the invasive stage that is capable of targeting recognition proteins, thus impairing host defenses.

In previous work conducted by our laboratory, *S* *. carpocapsae* virulence factors were identified from an EST library of the nematode during the parasitic stage and a set of serine protease inhibitor genes was detected [35]. ESTs encoding serine protease inhibitors are frequently identified during the infective stage of parasitic animal and plant nematodes, and this has led to the proposal that serine protease inhibitors participate in the host-pathogen arms race [39]. These data encouraged us to study the serine protease inhibitor Sc-KU-4, which was the most represented contig in the *S* *. carpocapsae* EST library. The full Sc-KU-4 cDNA shared identity with the secreted Kunitz inhibitors of the vertebrate parasitic nematodes *A* *. simplex* , *L. loa* and *A* *. suum* and was phylogenetically closest to that of *A* *. suum* . Sc-KU-4 belongs to the I2 BPTI–Kunitz family, as shown by full cDNA alignment in the MEROPS database. The Kunitz domain of Sc-KU-4 contains two conserved disulfide bridges (CysI - CysVI and CysIII - CysV) but lacks the third bridge (CysII - CysIV). The missing disulfide bridge was probably substituted by van-der-Waals interactions established between Gly36 and Gly61. These interactions might be important for the stabilization of the Sc-KU-4 binding site, owing to its allosteric properties [40]. The binding site residues in Sc-KU-4 differed from other Kunitz domains in particular hits, namely the predominance of polar residues (Gly, Ser, Thr, Asn), which may confer stability toward a broad range of trypsin-like enzymes. Furthermore, Sc-KU-4 presented a polar amino acid serine in the P1 binding site that is the pocket residue for binding trypsin, α-chymotrypsin and elastase-like enzymes [41,42]. In fact, assays performed with recombinant Sc-KU-4 showed that it effectively inhibited α-chymotrypsin and elastase through a competitive inhibitory mechanism, characteristic of the BPTI-kunitz family [43]. An identical spectrum of inhibition was observed in a Kunitz inhibitor released by the vertebrate parasitic nematode *A. ceylanicum*, which is proposed to participate in the depletion of the host immune system [44]. Interestingly, Sc-KU-4 exhibits 20% homology and shares structural features with the human bikunin (PDB 1BIK) protein, which inhibits leukocyte elastases, thereby modulating the activity of the inflammatory serine protease cascade and consequently impairing host defenses [45].

The analysis of Sc-KU-4 transcripts in the stages of the nematode life cycle and at different phases of parasitism showed that it was up-regulated during the invasion and installation of the parasite (L3 and L4), both of which occur while the insect is still alive, thus suggesting that the expression of Sc-KU-4 is somehow triggered in response to the host.

Serine proteases and serine protease inhibitors mediate different pathways of cellular and humoral responses in insects [4]. Recognition of invaders can activate intermediate steps consisting of serine proteases and their inhibitors that modulate the signal before activating the effectors, such as the Toll pathway [46], or can directly activate effectors of defense mechanisms, such as the induction of melanization [47]. Modulation of these recognition mechanisms is a strategy used by pathogens to impair host defenses. For example, the endoparasitoid *Venturia* *canescens* inhibits melanization in host hemolymph by releasing a trypsin-like serine protease inhibitor [48].

Our experiments demonstrate that Sc-KU-4 was able to deplete insect defenses by modulating the cellular and humoral immune reactions of *G* *. mellonella* through several mechanisms. First, Sc-KU-4 inhibited hemocyte aggregation that is the primary step in cellular encapsulation and requires the activation of granular cells and plasmatocytes and results in the entrapment of the invading pathogen [49]. In the presence of Sc-KU-4, despite the activation of hemocytes to an adherent state, few and small hemocyte aggregates were observed. Second, Sc-KU-4 did not interfere with clotting fiber activation but interferes with its structure, preventing the clot fibers to entrap foreign particles (beads), thus resulting in the reduced deposition of clotting materials. Third, Sc-KU-4 delayed PPO activation, despite the formation of final products was identical in the treated and non-treated assays. Taken together, these observations suggested that Sc-KU-4 may be interacting with proteins in the plasma.

Strongly bounded with Sc-KU-4, two plasmatic proteins were identified: a homolog of a masquerade-like protein (MSPH) and a homolog of a serine protease-like 1b (SPH-1). MSPH is a serine protease homolog that contains a trypsin-like domain at the C-terminal end in which the Ser in the catalytic site was replaced by a Gly, thus conferring the loss of its proteolytic activity. MSPH was demonstrated to be up-regulated in the plasma of Lepidoptera after parasitism [50] and to bind LPS and β-1,3-glucan in pathogens, promoting hemocyte adhesiveness [51]. SPH-1 has an identical mutation to that of MSPH in the catalytic triad [52]. SPH-1 was shown to form a complex in hemolymph with immulectin-2, prophenoloxidase and prophenoloxidase activating proteases, thus localizing the phenoloxidase activity in the surface of pathogens [53]. Interestingly, the clip-domain present in the N-terminal region of MSPH and SPH-1 was shown to play a role in the activation of proPO [54-56], whereas the C-terminal trypsin-like domain was involved in granulocyte adhesion, pattern recognition, and opsonization [51,52,57]. In accordance with our data, it is rational to hypothesize that Sc-KU-4 should target the catalytic site of SPHs, leaving the N-terminal region free, thus explaining why hemocyte adhesion and encapsulation were attained, while the activation of PO remained unaffected. Remarkably, two other PRP homologs, hemolin and apolipophorin III were interacting with Sc-KU-4.

MSPH and SPH targeted by Sc-KU-4 participate in the recognition of invading organisms as non-self thus were considered recognition proteins [51,58]. Recent investigations based on RNAi studies of genes encoding defense effector proteins and genes encoding the PRPs suggest that the PRP signaling pathway has a much greater impact on an insect’s survival after infection than does the effector gene pathway [58,59]. Thus, it is reasonable to assume that the ability of *S* *. carpocapsae* to evade host defenses in a wide number of insects [60] can be due to its ability to destroy a key pathway of invader recognition.

The identification of a virulence factor in a beneficial pathogen such as the EPN *S* *. carpocapsae* opens new avenues into the genetic improvement of this organism. Furthermore, it may be a useful tool in the study of host-pathogen interaction, using *S* *. carpocapsae* as a model, as recently proposed [61].

## Supporting Information

Table S1 **Insect plasma proteins targeted by Sc-KU-4 and identified by MS/MS.**
(DOCX)

Figure S1 **SDS-PAGE profiles of recombinant Sc-KU-4 produced under distinct conditions and purification steps.** **A**: Sc-KU-4 expression was examined in inclusion bodies and in soluble form after induction with 0.2 M IPTG at 20°C overnight (lanes 1 and 5); with 1 M IPTG at 20°C overnight (lanes 2 and 6); with 0.2 M IPTG at 37°C for 3 h (lanes 3 and 7) and with 1 M IPTG at 37°C for 3 h (lanes 4 and 8). Recombinant protein was expressed only in inclusion bodies and was present in the highest amounts in bacteria induced at 37°C (arrows in lanes 3 and 4). **B**: Sc-KU-4 expression was confirmed by Western blot using an anti-His6X-tag antibody. **C**: Recombinant Sc-KU-4 after enrichment with His-tag affinity chromatography (1) and a single 20 kDa band observed after purification with a MonoQ column (2). Proteins run on a 12% SDS-PAGE gel under reducing conditions and stained with Coomassie. MM is shown in kDa.
(TIF)
