# Supplementary material for: A Pathogenic Nematode Targets Recognition Proteins to Avoid Insect Defenses
Source: PLoS One. 2013 Sep 30;8(9):e75691. doi: 10.1371/journal.pone.0075691 (PMC3787073; doi:10.1371/journal.pone.0075691)
Supplement: Table S1 — Insect plasma proteins targeted by Sc-KU-4 and identified by MS/MS. (DOCX) [file pone.0075691.s001.docx]

**Table S1.**

| Protein identified | Acession number/Mw (kDa) | Protein Score^a^ | Total Ion Score^b^ | Obsrv. Mass  ± da | Error  ± da | MS/MS peptides^b^ |
| --- | --- | --- | --- | --- | --- | --- |
| Masquerade-like serine proteinase [Pieris rapae] | gi\|270298184/  45.192 | 138 | 93 | 1437.7074  2284.0903 | 0.0526  -0.1099 | FGEFPWMVAILK  MMNKLLVLIAVLSLAYCQDK |
| Serine proteinase-like protein 1b [Manduca sexta] | gi\|242351233/  45.594 | 108 | 98 | 1469.6765  2287.0784 | 0.0011  0.0401 | IEPVNENEPDGQK  LGRYFELHSSFMCAGGEPGR |
| Apolipophorin-3; | gi\|5915688/  20.441 | 571 | 512 | 1486.6982  1755.8251  1830.8779  1987.9965 | -0.0191  -0.0298  -0.0089  -0.033 | TFSEQLNAFTNSK  SAYDDFVKQAQEVQK  TFSEQLNAFTNSKDTK  EGSDSVLQQLNALASSLQK |
| Serine proteinase-like  [Bombyx mori] | gi\|114052256 / 43317.6 | 93 | 82 | 1430.6769  1478.678 | 0.0692  0.0641 | LAGLVAWGIGCGQK  DVPAVYANVARMR |
| Imaginal disc growth factor  [Biston betularia] | gi\|308512729/ 21.997 | 236 | 222 | 861.3851  3008.415 | -0.0403  -0.0486 | FGTYAFR  TWKLDSDSEIAGVPPLHADGPGEAGPYTK |
| Immulectin-2 [Manduca sexta] | gi\|259493819 / 48.168 | 107 | 95 | 866.4487  2576.2139 | -0.029  -0.06 | GMRPHLR  TFGTTPVDDKEAEHREGFTALVR |

a: The protein score probability limit (where P < 0.05) is 85.

b: peptides with confidence interval above 95% were considered.
