# Supplementary figures and images for: A Pathogenic Nematode Targets Recognition Proteins to Avoid Insect Defenses
Source: PLoS One. 2013 Sep 30;8(9):e75691. doi: 10.1371/journal.pone.0075691 (PMC3787073; doi:10.1371/journal.pone.0075691)

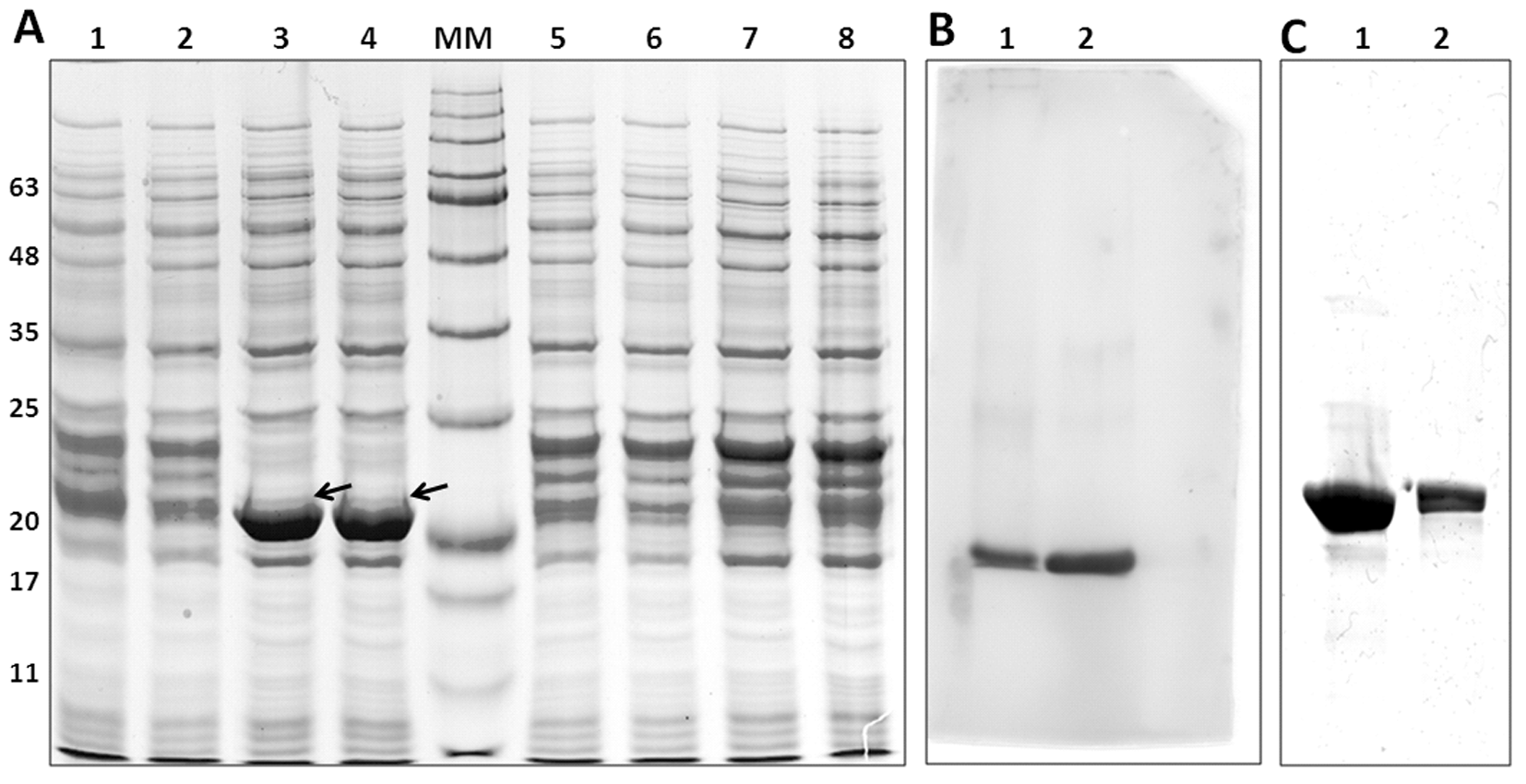

Supplement: Figure S1 — SDS-PAGE profiles of recombinant Sc-KU-4 produced under distinct conditions and purification steps. A: Sc-KU-4 expression was examined in inclusion bodies and in soluble form after induction with 0.2 M IPTG at 20°C overnight (lanes 1 and 5); with 1 M IPTG at 20°C overnight (lanes 2 and 6); with 0.2 M IPTG at 37°C for 3 h (lanes 3 and 7) and with 1 M IPTG at 37°C for 3 h (lanes 4 and 8). Recombinant protein was expressed only in inclusion bodies and was present in the highest amounts in bacteria induced at 37°C (arrows in lanes 3 and 4). B: Sc-KU-4 expression was confirmed by Western blot using an anti-His6X-tag antibody. C: Recombinant Sc-KU-4 after enrichment with His-tag affinity chromatography (1) and a single 20 kDa band observed after purification with a MonoQ column (2). Proteins run on a 12% SDS-PAGE gel under reducing conditions and stained with Coomassie. MM is shown in kDa. (TIF) [file pone.0075691.s002.tif]
